# Molecular dynamics modeling framework for overcoming nanoshape retention limits of imprint lithography

**DOI:** 10.1038/s41378-018-0007-4

**Published:** 2018-04-23

**Authors:** Anshuman Cherala, S. V. Sreenivasan

**Affiliations:** NASCENT Center, MERB 1.206E, Pickle Research Campus, University of Texas, 10100 Burnet Road, Austin, TX 78758 USA

## Abstract

Complex nanoshaped structures (nanoshape structures here are defined as shapes enabled by sharp corners with radius of curvature <5 nm) have been shown to enable emerging nanoscale applications in energy, electronics, optics, and medicine. This nanoshaped fabrication at high throughput is well beyond the capabilities of advanced optical lithography. While the highest-resolution e-beam processes (Gaussian beam tools with non-chemically amplified resists) can achieve <5 nm resolution, this is only available at very low throughputs. Large-area e-beam processes, needed for photomasks and imprint templates, are limited to ~18 nm half-pitch lines and spaces and ~20 nm half-pitch hole patterns. Using nanoimprint lithography, we have previously demonstrated the ability to fabricate precise diamond-like nanoshapes with ~3 nm radius corners over large areas. An exemplary shaped silicon nanowire ultracapacitor device was fabricated with these nanoshaped structures, wherein the half-pitch was 100 nm. The device significantly exceeded standard nanowire capacitor performance (by 90%) due to relative increase in surface area per unit projected area, enabled by the nanoshape. Going beyond the previous work, in this paper we explore the scaling of these nanoshaped structures to 10 nm half-pitch and below. At these scales a new “shape retention” resolution limit is observed due to polymer relaxation in imprint resists, which cannot be predicted with a linear elastic continuum model. An all-atom molecular dynamics model of the nanoshape structure was developed here to study this shape retention phenomenon and accurately predict the polymer relaxation. The atomistic framework is an essential modeling and design tool to extend the capability of imprint lithography to sub-10 nm nanoshapes. This framework has been used here to propose process refinements that maximize shape retention, and design template assist features (design for nanoshape retention) to achieve targeted nanoshapes.

## Nanoscale patterning: modeling framework to achieve targeted nanoshapes

A molecular dynamics (MD) model was developed to capture the shape retention behavior of polymers at the nanometer scale. Complex nanoshape (nanometer-scale) structures facilitate the development of applications in a great diversity of fields. However, even the best e-beam lithography processes achieve resolutions of only around 20 nm with long range order over large areas at low processing rates. A team comprised of A. Cherala and S.V. Sreenivasan of the University of Texas was able to extend the capability of imprint lithography to nanoshapes under 10 nm. The team believes that these sophisticated models allow compensation of parasitic behavior in polymers, thereby enabling shape retention for desired nanoshape structures.

## Introduction

New applications in varied fields such as energy storage^1^, nanoscale photonics^2^, plasmonic nanostructures^3^, multi-bit magnetic memory^4^, terabit per square inch magnetic recording^5^, and bio-nanoparticles^6, 7^ require high-throughput patterning with complex shape control at the nanoscale. Shaped nanopatterns exhibit novel optical, mechanical, and morphological properties that are exploited in various ways by these emerging fields.

The state-of-the-art form of optical lithography—193 nm immersion (193i) lithography—has plateaued at a resolution of approximately 38 nm half-pitch for gratings, and ~50 nm half-pitch for more complex structures. Higher-resolution large-area patterns are currently manufactured by complementing photolithography with self-aligned double patterning (SADP) and multiple lithography-etch steps. Directed self-assembly (DSA) is also being explored, however both SADP and DSA are primarily restricted to periodic features^8–10^. While the highest-resolution e-beam processes (Gaussian beam tools with non-chemically amplified resists) can achieve <5 nm resolution^11^, this is only available at very low throughputs. Large-area e-beam fabrication using variable shape beam tools, needed for photomasks and imprint templates, are limited to ~18 nm half-pitch lines and spaces^12^ and ~20 nm half-pitch hole patterns^13^. Other techniques such as extreme ultraviolet (UV) lithography, and multiple e-beam lithography, face fundamental challenges such as lack of light source, line edge roughness, and low throughput^14, 15^.

**Fig. 1:**
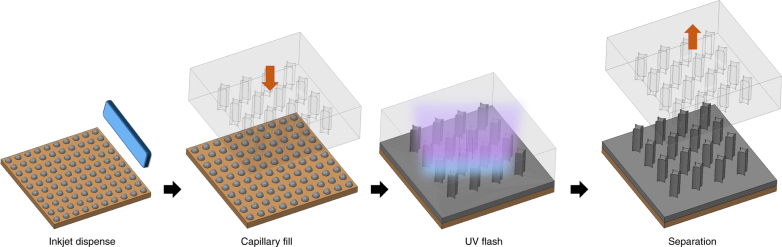
Jet and Flash Imprint Lithography

Imprint lithography has demonstrated large-area patterning at sub-10 nm half-pitch with the capability to pattern typical lithographic structures such as lines, gratings, dot arrays, etc.^16–19^. Due to its near molecular level of resolution over large areas and its progress in scalability, Jet and Flash Imprint Lithography (J-FIL) is a viable candidate for manufacturing sub-20 nm patterns in semiconductor devices^20^ and for sub-10 nm patterns in hard disks^21^. In J-FIL, a low-viscosity resist is deposited onto the substrate using an inkjet dispenser. This dispensing technique has been chosen in J-FIL to match the distribution of resist to the pattern density variation in the template, which enables high-throughput patterning of arbitrary structures. The patterned template is then lowered onto the dispensed material on the substrate so that the relief patterns are filled by capillary action. The resist material, which is an acrylate-based multi-component formulation, is then crosslinked under UV radiation. Finally, the template is removed leaving a patterned resist on the substrate (Fig. 1).

We have recently demonstrated a 90% increase in ultracapacitor performance, made with J-FIL. The device is fabricated with shaped silicon nanowires having a diamond-like cross section, compared to a conventional circular cross section^1^. A further 10× increase in performance is possible with lithographic scaling to 10 nm half-pitch shaped nanowires.

**Fig. 2:**
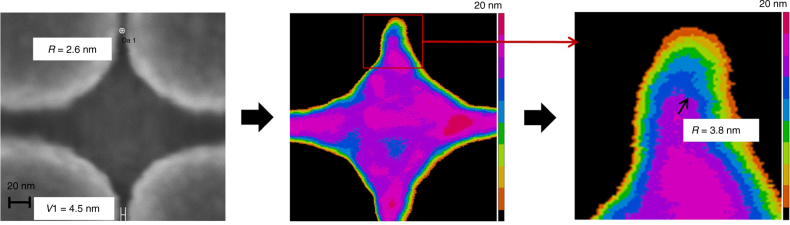
SEM and AFM images show 2.6 nm radius corner on template (left) is replicated in the resist (center) and measured at 3.8 nm radius with AFM (right). The SEM image shows the sharp corner measured at the base of the pillars on the template. The AFM images show the corresponding top of the feature in imprint resist with each color representing a 2 nm horizontal slice; this location represents the ultra-sharp corner of the imprinted resist

As part of the ultracapacitor fabrication process, careful measurements were performed to compare the diamond feature on the imprint template and the replicated resist. It was observed that the radius of the sharp corner on the template was larger when measured on the imprinted resist feature (Fig. 2). A finite element model used to estimate the resist shrinkage did not capture this behavior. The model in fact predicted the opposite trend while accurately predicting changes in the larger dimensions of the diamond. The finite element model assumes a uniform elastic modulus and Poisson’s ratio, which are model inputs, where as it has been reported in the literature that the bulk properties of materials, especially polymers lose homogeneity for dimensions below few monomer lengths^22^. We believe this discrepancy is a key contributor to the failure of this model to capture the corner relaxation behavior.

This failure led to the search for a more sophisticated model for the sub-5 nm-length scales of interest for nanoshaped structures. Such a model would be an essential design tool, for example, to enable scaling of the ultracapacitor density with 10 nm half-pitch shaped structures as mentioned earlier. Section “Atomic model of resist nanoshape” describes the molecular dynamics (MD) model developed for the nanoshape resist structure. Section “Model validation” presents model validation results. Shape retention as a function of geometry and feature size are discussed in section “Shape retention in nanoshaped resist structures.” Ideas for optimizing shape retention are presented in section “Design for nanoshape retention.”

## Atomic model of resist nanoshape

Due to the aforementioned failure of the continuum mechanics-based model, an all-atom MD framework was chosen to model the imprint resist, after conducting a survey of models in the nanoscale regime. The atomic model is expected to accurately capture polymer resist behavior below 5 nm and predict a wide range of properties with minimal a priori assumptions regarding material properties, while still being computationally affordable. Bulk material properties will in fact be an output from this MD model. Various degrees of so-called coarse graining can be done in MD by lumping several atoms or clusters into one effective mass^23^. The coarse-grained models reduce the number of masses and therefore reduce computational cost. We chose not to use coarse graining but instead model each atom in the resist monomer molecules, since the dimensions of interest for nanoshape retention in resist are of the order of the size of a single molecule of monomer.

In all-atom MD, atoms are modeled as point particles with mass and optionally electric charge. The masses assigned are the atomic weights of the respective atom types. Even neutral molecules have partially charged atoms due to electron clouds getting pulled toward the heavier nuclei. Atom connectivity is defined by the topology of the molecule. Bonded connections are modeled as “springs” (linear and nonlinear). Non-bonded interactions are divided into van der Waals and electrostatic components. The van der Waals interactions is modeling using a Lennard-Jones potential function and the electrostatic component with a Coulomb potential. The spring properties are part of the so-called forcefield obtained from quantum mechanics (ab initio methods). Atoms obey Newton’s laws of mechanics when subject to forces dictated by this forcefield. Initial atom velocities are a function of temperature as shown in Eq. 2. A Gaussian distribution of speed (with net momentum = 0, to prevent the entire system from drifting) is assigned to the atoms.

1 $${\mathrm{KE}}_{{\mathrm{avg}}}{\mathrm{ = }}\overline {\left[ {\frac{1}{2}mv^2} \right]} {\mathrm{ = }}\frac{3}{2}k_{\mathrm{B}}T$$

where,

KE_avg_ = average kinetic energy of the system of particles

*m* = mass of particle

*v* = velocity of particle

*k*_B_ = Boltzmann’s constant

*T* = temperature

Note that the forcefield (from quantum mechanics), atom positions, and velocity profile (from statistical mechanics/kinetic theory) are the only inputs to the model. No other material properties are input, rather they are derived from the MD model. This capability of MD is powerful at the nanoscale.

Time stepping is typically performed by a velocity Verlet algorithm. It should be noted here that the while other time-stepping algorithms like fourth-order Runge-Kutta methods exist, the velocity Verlet algorithm has been shown to track the “ghost” Hamiltonian of the system for extended time periods thus guaranteeing stability while being computationally affordable.

Pressure is defined at the atomic scale by a virial expansion as shown in Eq. 2.

2 $$P{\mathrm{ = }}\frac{{Nk_{\mathrm{B}}T}}{V} + \frac{{\mathop {\sum }\nolimits_i^N x_i.f_i}}{{{\mathrm{d}}V}}$$

where,

*P* = pressure

*N* = number of atoms/particles in the system

*V* = volume of system

*x*_*i*_ = position of atom *i*

*f*_*i*_ = force on atom *i*

Common MD “fixes” or thermodynamic control actions include barostatting by adjusting simulation box volume to achieve pressure setpoint and thermostatting by adjusting particle velocities to achieve temperature setpoints.

**Fig. 3:**
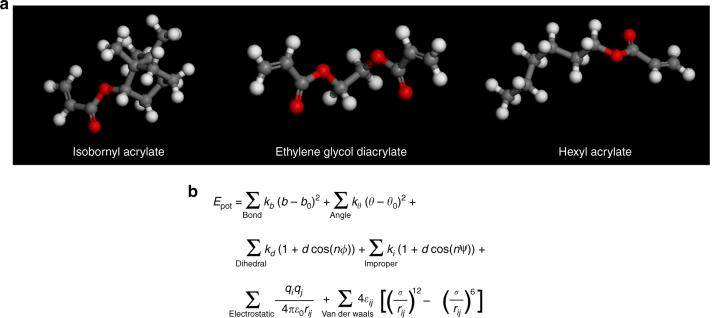
MD resist model. **a** Imprint resist components. **b** CVFF forcefield functional form

The imprint resist formulation was taken from the patent literature^24^. The resist consists of three acrylate molecules namely, hexyl acrylate (54% w/w), isobornyl acrylate (25% w/w), and ethylene glycol diacrylate (19% w/w) as the crosslinker, as shown in Fig. 3a. The atoms and molecule models were created in Accelrys Materials Studio software. The topology of the molecule, atom types, bond connectivity, and type can be selected and sketched in Materials Studio and the monomer allowed to reach a minimum energy configuration. A volume of monomer resist is then generated based on the molecule packing routine of that package. The Consistent Valence Force Field, originally developed to study organic molecules, is used to model the intermolecular interactions^25^. The functional form of the forcefield is shown in Fig. 3b. Four bonded interactions namely bond, angle, dihedral, and improper interactions are incorporated. Partial charges are assigned to dissimilar bonded atoms. Non-bonded interactions include a Lennard-Jones potential for the van der Waals interaction and a Coulomb interaction for the partial charges. The cutoff distance for non-bonded interactions is set to 9.5 Å. The resist system is then imported into the Large-scale Atomic/Molecular Massively Parallel Simulator (LAMMPS) MD software^26^. LAMMPS is a popular open-source MD software developed and supported by Sandia National Labs. Model visualization is done in JMOL and VMD software packages. Please refer to the supplementary file for a detailed molecule creation procedure and sample LAMMPS scripts.

## Model validation

MD polymer models may be validated by estimating the bulk resist modulus, strength, and glass transition temperature. The liquid monomer model imported from Materials Studio is first checked for accuracy by comparing total system energy and the various potential energy components namely bond, angle, dihedral, improper, and van der Waal energies, respectively, to ensure the atom locations and force interactions were imported correctly into LAMMPS.

The bulk system consists of a 150 × 150 × 130 Å volume with around 286 000 atoms. The system is first equilibrated in an isothermal-isobaric (NPT) ensemble at 298 K and 1 atm. pressure with a Nose-Hoover-style thermostat. The time integration is done by the velocity Verlet algorithm at 1 fs intervals. Acrylate polymerization is then performed based on a cutoff distance between two double-bond carbons. More specifically, the bond creation step looks for double-bonded carbon atoms present within a certain radius. The closest pair is then converted to a single bond and the appropriate molecule topology and bond parameters are updated. This procedure is repeated till there are no more bonds possible. It was observed that the extent of polymerization was around 85–90%, which is consistent with numbers reported in literature^27^.

The elastic constants of the polymer are estimated by straining the block of material in the three axial and shear directions. The change in stress tensor for each uniform strain component is used to calculate the elements of the 6 × 6 elastic matrix. The stress-strain relationship and form of the elastic matrix for isotropic materials are shown in Eq. 3. The values obtained for the polymer resist are shown in Eq. 4. The Young’s modulus and Poisson’s ratio estimated from the elastic constant is 1.1 GPa and 0.42, respectively. These numbers are consistent with numbers reported in the literature for similar materials like PMMA.

3 $${\left[ {\begin{array}{*{20}{c}} {\sigma _{11}} \\ {\sigma _{22}} \\ {\sigma _{33}} \\ {\sigma _{23}} \\ {\sigma _{13}} \\ {\sigma _{12}} \end{array}} \right]{\mathrm{ = }}\frac{E}{{(1 + \upsilon )(1 - 2\upsilon )}}\left[ {\begin{array}{*{20}{c}} {1 - \upsilon } \\ \upsilon \\ \upsilon \\ 0 \\ 0 \\ 0 \end{array}\begin{array}{*{20}{c}} \upsilon \\ {1 - \upsilon } \\ \upsilon \\ 0 \\ 0 \\ 0 \end{array}\begin{array}{*{20}{c}} \upsilon \\ \upsilon \\ {1 - \upsilon } \\ 0 \\ 0 \\ 0 \end{array}\begin{array}{*{20}{c}} 0 \\ 0 \\ 0 \\ {\frac{{1 - 2\upsilon }}{2}} \\ 0 \\ 0 \end{array}\begin{array}{*{20}{c}} 0 \\ 0 \\ 0 \\ 0 \\ {\frac{{1 - 2\upsilon }}{2}} \\ 0 \end{array}\begin{array}{*{20}{c}} 0 \\ 0 \\ 0 \\ 0 \\ 0 \\ {\frac{{1 - 2\upsilon }}{2}} \end{array}} \right]\left[ {\begin{array}{*{20}{c}} {\varepsilon _{11}} \\ {\varepsilon _{22}} \\ {\varepsilon _{33}} \\ {\varepsilon _{23}} \\ {\varepsilon _{13}} \\ {\varepsilon _{12}} \end{array}} \right]}$$

4 $$\begin{array}{l}\frac{E}{{\left( {1 + \upsilon } \right)(1 - 2\upsilon )}}\left[ {\begin{array}{*{20}{c}} {1 - \upsilon } \\ \upsilon \\ \upsilon \\ 0 \\ 0 \\ 0 \end{array}\begin{array}{*{20}{c}} \upsilon \\ {1 - \upsilon } \\ \upsilon \\ 0 \\ 0 \\ 0 \end{array}\begin{array}{*{20}{c}} \upsilon \\ \upsilon \\ {1 - \upsilon } \\ 0 \\ 0 \\ 0 \end{array}\begin{array}{*{20}{c}} 0 \\ 0 \\ 0 \\ {\frac{{1 - 2\upsilon }}{2}} \\ 0 \\ 0 \end{array}\begin{array}{*{20}{c}} 0 \\ 0 \\ 0 \\ 0 \\ {\frac{{1 - 2\upsilon }}{2}} \\ 0 \end{array}\begin{array}{*{20}{c}} 0 \\ 0 \\ 0 \\ 0 \\ 0 \\ {\frac{{1 - 2\upsilon }}{2}} \end{array}} \right]\\ = \left[ {\begin{array}{*{20}{c}} {2.77} \\ {1.98} \\ {1.98} \\ {0.01} \\ { - 0.01} \\ {0.004} \end{array}\begin{array}{*{20}{c}} {1.98} \\ {2.74} \\ {1.97} \\ { - 0.008} \\ {0.007} \\ { - 0.01} \end{array}\begin{array}{*{20}{c}} {1.98} \\ {1.97} \\ {2.74} \\ {0.009} \\ {0.012} \\ { - 0.006} \end{array}\begin{array}{*{20}{c}} {0.01} \\ { - 0.008} \\ {0.009} \\ {0.02} \\ 0 \\ {0.36} \end{array}\begin{array}{*{20}{c}} { - 0.01} \\ {0.007} \\ {0.012} \\ {0.006} \\ {0.35} \\ { - 0.01} \end{array}\begin{array}{*{20}{c}} {0.004} \\ { - 0.01} \\ { - 0.006} \\ {0.36} \\ { - 0.01} \\ { - 0.001} \end{array}} \right]\end{array}$$

**Fig. 4:**
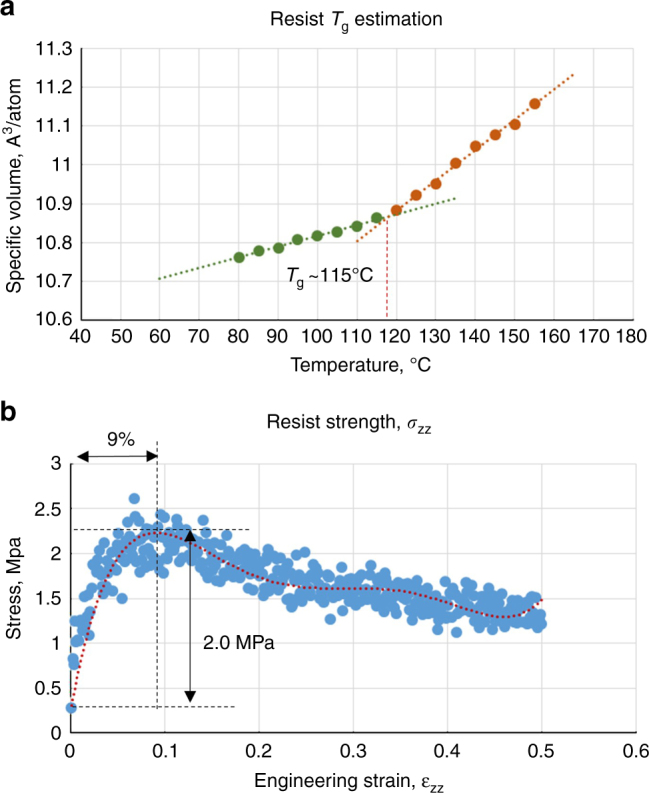
Resist model validation. **a** Resist glass transition temperature estimation. **b** Resist strength estimation by simulated tensile test

Polymers are unique in exhibiting a significant change in mechanical properties at the glass transition temperature, *T*_g_. This temperature is estimated by calculating the average volume over a range of temperatures. Figure 4a shows the change in specific volume with temperature. *T*_g_ is estimated as the intersection point of the two linear sections of the curve. The value for this polymer system is estimated around 115 °C. This compares well for example, with *T*_g_ for PMMA reported around 105 °C in the literature.

Resist strength is estimated by simulating a tensile test with uniaxial strain and observing peak stress. Figure 4b shows stress from 0 to 50% strain. The yield stress is estimated at around 2.0 MPa for 9% elongation based of a polynomial trend line fit. The model validation simulations are computationally very demanding and therefore most of the runs were done on the Texas Advanced Computing Center supercomputers.

## Shape retention in nanoshaped resist structures

**Fig. 5:**
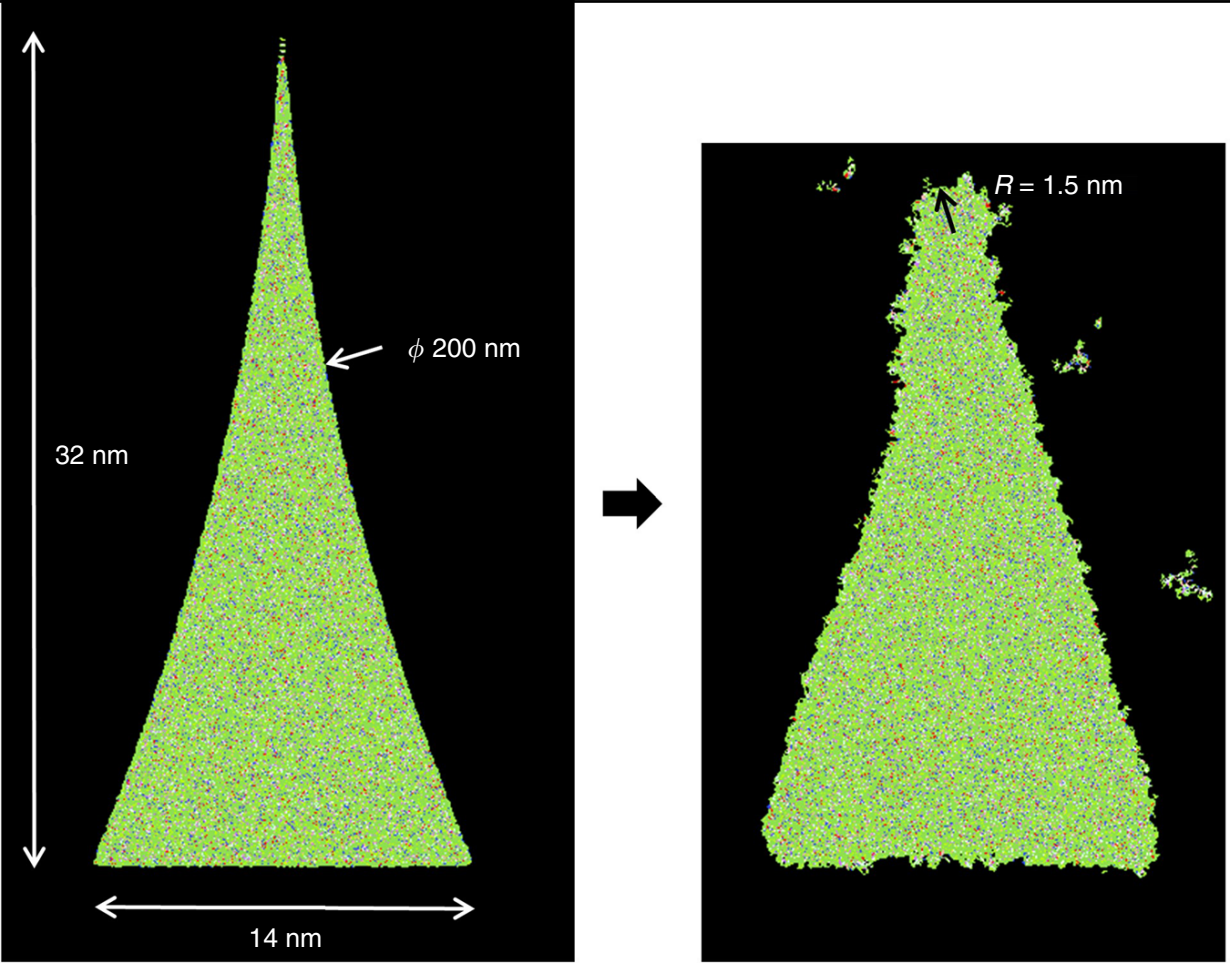
Atomically sharp corner of 200 nm diamond-like nanoshape (left) and after 50 ps relaxation (right)

After having rigorously validated the atomic resist model, it can be used to predict shape retention. The 200 nm diamond-like nanoshape structure is partially modeled (due to size limitations) in MD as an ideally sharp corner and allowed to relax for 50 ps. The ideal geometric shapes of the imprint template are created in resist by defining primitive shapes like cylinders or rectangular boxes, and retaining only the molecules inside or outside of the chosen regions in the system as required. LAMMPS has the capability to create a van der Waals interaction between the walls of the chosen region and the atoms, so that the walls gently repel the atoms as they get close to the region boundaries. This feature and a careful selection of timestep prevents atoms from moving out of this simulated template nanoshape. After the system is polymerized in this state, the region is removed from the system and the crosslinked resist is allowed to move without restriction. As can be seen in Fig. 5, the model is able to qualitatively predict relaxation observed in experiment.

**Fig. 6:**
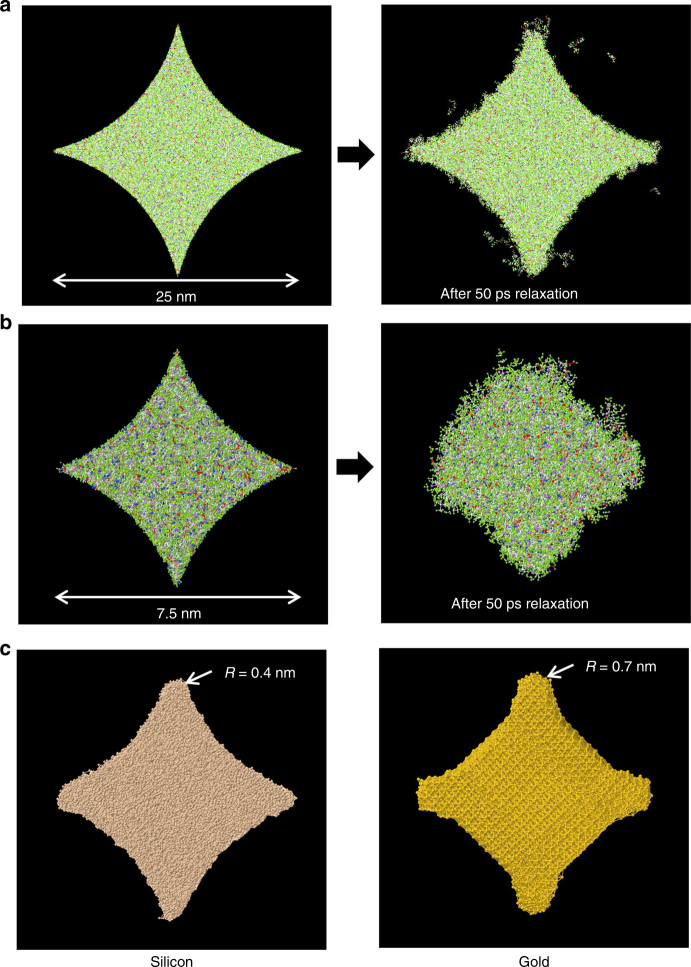
Nanoshape retention study. **a** Shape retention in 25 nm diamond-like nanoshape resist structure. **b** Failure of shape retention in 7.5 nm diamond-like nanoshape resist structure. **c** Same geometry in silicon and gold show significantly better shape retention

Having thus developed a model that is further validated with experimental data, we can investigate shape retention capability of the resist at the dimensions of interest (below 25 nm). To that end, a 25 nm and a 7.5 nm diamonds are modeled. Figure 6 shows the shape retention after 50 ps. As can be seen, the 25 nm diamond retains shape except for the sharp corners relaxing and becoming rounded. The 7.5 nm diamond on the other hand fails to retain the original desired shape. Figure 6c also shows the material dependence on shape retention. The same 7.5 nm diamond geometry is modeled in crystalline silicon with a diamond lattice using the classical Stillinger-Webber multibody potential^28^ and fcc crystalline gold using the EAM potential^29^. The two materials retain shape significantly better than polymer resist. The radius of the corners is measured at 0.4 nm for silicon and 0.7 nm for gold. This shows that the limiting step in the nanoshape fabrication process is shape retention in polymer resist. If the shape is successfully retained in the resist, there should be no problem retaining shape in the underlying hard mask and substrate layers.

Thus, the MD model developed here can be used for optimizing resist material composition, template design, identifying critical dimensions for shape retention, and modeling different materials within a uniform framework. More specifically, for the ultracapacitor application, the model suggests that the 25 nm diamond structures are viable, whereas the 7.5 nm structures are not. This kind of information and analysis, from a first principles-based, validated model is a valuable design tool for template and process design. The modeling data minimizes trial and error and costly design of experiments, which would otherwise be required to characterize the nanoshape viability. Some ideas to overcome the size limitation for nanoshape imprinting are discussed in the next section.

## Design for nanoshape retention

Three ideas to improve shape retention are discussed in this section namely tone inversion, addition of compensating sub-resolution features to the template, and exploiting the properties of subsequent reactive ion etch (RIE) etching.

### Tone inversion

**Fig. 7:**
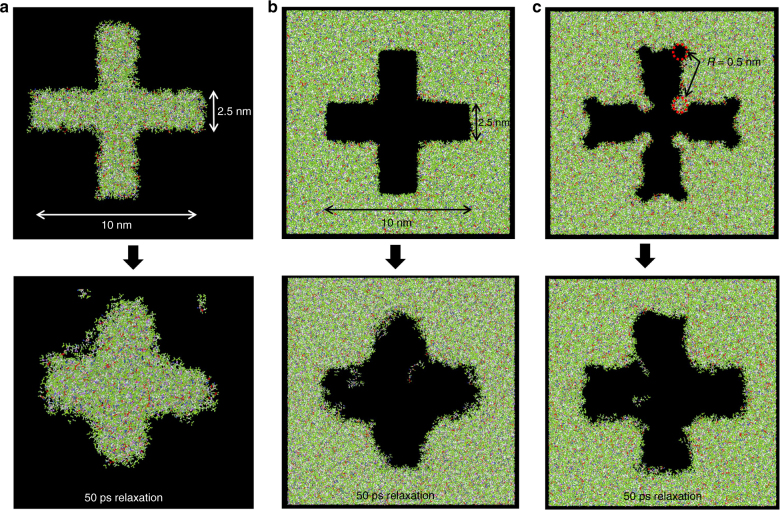
Design for Nanoshape Retention (DNR): Tone Inversion and addition of sub-resolution shape retention enhancement features. **a** A 10×2.5 nm cross nanoshape resist feature. **b** 10×2.5 nm cross nanoshape resist feature hole (reverse tone). **c** Reverse tone cross feature with sub-resolution shape retention enhancement

Imprint lithography has been practiced in reverse tone, notably in bit patterned media applications and is well established^30^. For nanoshape imprinting, this aspect is very useful and once the critical dimension (for shape retention) for a given geometry is identified following the procedure discussed in the previous section, sub-CD features should be pursued in the reverse tone. This is exemplified in Fig. 7a, b where a cross nanoshape of size 10 × 2.5 nm is shown in both tones. Cross-shaped nanostructures have potential applications in new memory devices like spin transfer torque RAM^4, 31^. As can be seen, shape retention in the cross feature is very poor. The cross shape retention is significantly better in the reverse tone (cross-shaped hole).

### Addition of sub-resolution features

As can also be seen, while the reverse tone cross retains shape better, it loses original shape due to geometry effects and resist relaxation. Addition of sub-resolution design for nanoshape retention (DNR) features can compensate for this and maintain target cross shape significantly better as shown in Fig. 7c. As with other models, the system was run for 50 ps. The system energy was tracked and observed to reach steady state after around 5 ps. This indicates that the system has reached equilibrium after 50 ps. These DNR features are similar to optical proximity correction (OPC) patterns that are well-known in optical lithography where they are used to compensate for diffraction and other optical effects at the nanoscale. Here we have demonstrated the design and use of OPC-like features for nanoshape imprint lithography to enhance shape retention by compensating for resist shrinkage, relaxation, and other geometry-induced effects. Templates can be fabricated with these enhancing features, for example, with STM tip-based methods^32^.

### Etch compensation

**Fig. 8:**
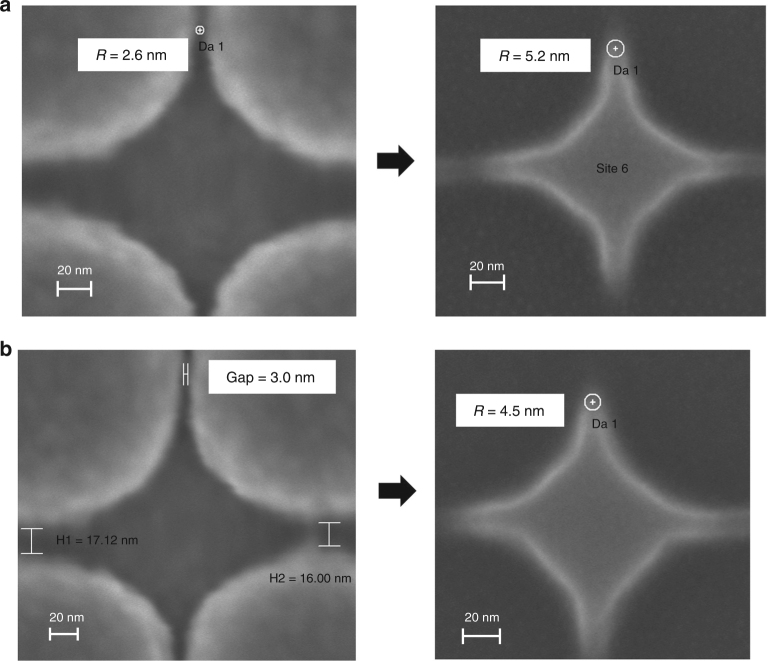
RIE etch based DNR. **a** SEM image of diamond-like shape template feature (left) with 2.6 nm radius corner and corresponding feature after RIE etch (right) into silicon oxide with 5.2 nm radius corner. **b** SEM image of diamond-like shape template feature (left) with 3.0 nm bridge gap and corresponding feature after RIE etch (right) into silicon oxide with 4.5 nm radius corner

Another aspect of the pattern transfer process that can be exploited is the fact that the RIE process that transfers the shape into the underlying substrate tends to have a small isotropic etch rate. This can be used, for example, by designing the template to have a thin connection between adjacent diamond features as shown in Fig. 8b. This gap creates a narrow bridge in the imprinted resist. The subsequent etching process breaks down the 3 nm bridge, leaving a sharper corner when compared to starting with an isolated diamond feature. This phenomenon was exploited with the diamond shape. Figure 8a, b shows an isolated diamond with 2.6 nm corner and diamond with a thin connecting bridge (left images) and the corresponding structures after etching into the underlying oxide layer (right images). As can be seen, the bridged diamond shows the sharper corner after RIE, due to the effect explained above.

## Conclusion

Nanoshaped structures enable emerging technologies across different applications as recently demonstrated by the ultracapacitor device fabrication. A new shape retention limit was identified when exploring the scaling of these nanoshaped structures to 10 nm half-pitch and below due to polymer relaxation in imprint resists. A gap was thus identified in the modeling framework required to complement the fabrication and process design for these structures. All-atom MD was found to be a very good modeling fit for nanoshape lithography. The atomic modeling scheme is sophisticated enough to capture the intricate behavior of polymers and other materials of interest at the nanometer scale and at the same time predict bulk properties, while being computationally affordable. The model can be used to design imprint resists with the desired material properties, predict shape retention, and select imprint tone. DNR techniques including imprint template design optimization can be performed with this model by adding shape-enhancing sub-resolution features similar to OPC features for optical lithography masks.

## Electronic supplementary material

Material Studio & LAMMPS Molecule Creation and MD Procedures
