# Supplementary material for: Molecular dynamics modeling framework for overcoming nanoshape retention limits of imprint lithography
Source: Microsyst Nanoeng. 2018 Apr 23;4:3. doi: 10.1038/s41378-018-0007-4 (PMC6220163; doi:10.1038/s41378-018-0007-4)
Supplement: Supplementary file 1 — Material Studio & LAMMPS Molecule Creation and MD Procedures [file 41378_2018_7_MOESM1_ESM.pdf]

Material Studio & LAMMPS Molecule Creation and MD Procedures

(Supplementary material for article titled, "Molecular Dynamics Modeling Framework for Overcoming Nanoshape Retention Limits of Imprint Lithography")

This document details the creation of an organic molecule in Materials Studio (MS) software sold by Accelrys (now Biovia) and import into LAMMPS for use in Molecular Dynamics simulations. The MS software has a very easy to use GUI that lets the user select atom types and sketch a molecule with the required bonds and topology. MS also has its own MD solver packages and custom forcefields. Each component of the resist is created in this way and a minimum energy structure generated. MS also has a volume packing routine that allows for creation of a multi component block of material with a required density and weight percentages.

A detailed step-by-step procedure to create a molecule in MS, export to Large-scale Atomic/ Molecular Massively Parallel Simulator (LAMMPS) and run a simple MD simulation is explained next. LAMMPS is a popular open source MD software developed and supported by Sandia National Labs. Model visualization is done in JMOL and VMD software packages.

The example molecule chosen is hexyl acrylate.

Step 1: Creating the molecule.

Open MS and select *New > 3D Atomistic Document* from the drop down menu. This creates a blank document. The molecule library of MS contains the most commonly used atom types. Select and sketch the atoms in your molecule. Bond topologies can be defined between the sketched atoms and atom types. Note: Stick figures are default display style in MS. Right click on the sketched stick figure to change display style to ball and stick type to match the figures shown in this document. The distances and angles between atoms is not important in the sketching step since the minimization step later will re-position the atoms to a minimum energy state.

Step 2: Selecting the forcefield

After the molecule is sketched, save the file and open *Modules* > *Discover* > *Setup*. Here the forcefield and other parameters related to forcefield setup can be chosen. A suitable forcefield from a library is chosen to perform the energy minimization and the dynamic timestepping.

Step 3: Minimization

Open *Modules* > *Discover* > *Minimizer* to setup a minimization run and execute the minimization. This will create a new folder and the minimized configuration of the molecule is stored here. As part of the minimization, the different potential energy components from bonded and non-bonded interactions are output. These values will need to match with the energy components generated by LAMMPS after importing.

Step 4: Exporting the file

Molecules are exported to a *.car* format by *File* > *Export*. Two files with *.car* and *.mdf* extension will be created upon exporting.

Step 5: To create a multi-molecule volume of material, repeat Step 1-3 for each molecule in your system and save the minimized molecule files.

Step 6: Creating a volume of resist

To create a volume of monomer resist from the different molecules, create another 3D Atomistic Document like in step 1. Create a volume to pack the molecules in with *Build* > *Crystals* > *Build Crystal*. This opens a dialog box, type in the dimensions of box, for example 50A x 50A x 50A. Next open *Modules* > *Amorphous Cell* > *Calculation.* This opens a pop up window. Select *packing* option in the task list (other options are *construction* for creating a polymer volume and *confined layer*). Here the different component molecules created in step 4 can be selected along with their Mole Ratio/ weight percentage in the mixture. The target density of the multicomponent resist and other forcefield related parameters maybe selected. Click *run* to start the packing algorithm. The packing may take anywhere from 10 minutes to several hours depending on the size of the volume. Once the volume is populated, save the file. The output of the volume packing also gives some statistics about the energies of the different bonded and non-bonded interactions.

These will need to match the numbers generated by LAMMPS later. This file can also be exported to a *.car* format.

Figure 1 shows a screenshot of a sketched hexyl acrylate molecule.

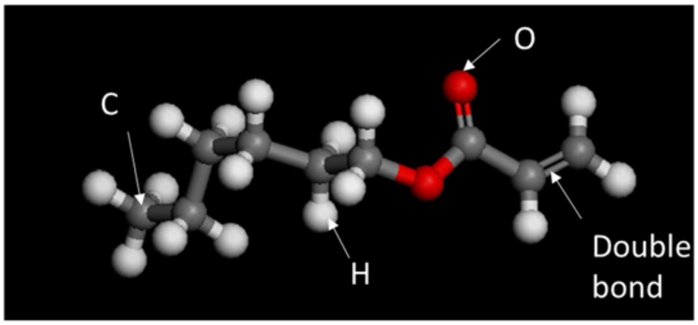

Figure 1: Hexyl acrylate molecule

Step 7: Importing into LAMMPS.

Find the forcefield data file, usually located in *C:\Program Files (x86)\Accelrys\Materials Studio\share\Discover\res*. The file name will be something like *cvff.frc*. This file along with the *.car* & *.mdf* files are required to convert the MS data to LAMMPS compatible data. LAMMPS package can be downloaded from *http://lammps.sandia.gov/*. The package has a data convertor executable called *msi2lmp.exe*. The inputs for this executable are the *.car* & *.mdf* data files and the forcefield file. Please refer to instructions in the LAMMPS package about how to use *msi2lmp.exe*.

Step 8: A data file with extension *.data* is created after successfully running *msi2lmp.exe*. This data file contains all the information about the system including atom positions, charges, bond topology, and force field parameters. This file is directly read into the LAMMPS script. A sample *.data* file of a hexyl acrylate molecule is shown below.

```
LAMMPS data file. msi2lmp v3.9.7 / 24 Oct 2015 / CGCMM for
nhexyl_acrylate_monomer

      27 atoms
      26 bonds
      46 angles
      58 dihedrals
       3 impropers
```

```
   7 atom types
  10 bond types
  17 angle types
  19 dihedral types
   3 improper types

    -28.554717730     23.589615901 xlo xhi
    -22.021477635     22.578568185 ylo yhi
    -22.346226820     23.182419172 zlo zhi

Masses

   1  12.011150 # c3
   2  12.011150 # c2
   3  15.999400 # o
   4  12.011150 # c'
   5  15.999400 # o'
   6  12.011150 # c=
   7   1.007970 # h

Pair Coeffs # lj/cut/coul/long

   1   0.0389999952   3.8754094636 # c3
   2   0.0389999952   3.8754094636 # c2
   3   0.2280000124   2.8597848722 # o
   4   0.1479999981   3.6170487995 # c'
   5   0.2280000124   2.8597848722 # o'
   6   0.1479999981   3.6170487995 # c=
   7   0.0380000011   2.4499714540 # h

Bond Coeffs # harmonic

   1   322.7158     1.5260 # c3-c2
   2   340.6175     1.1050 # c3-h
   3   322.7158     1.5260 # c2-c2
   4   340.6175     1.1050 # c2-h
   5   273.2000     1.4250 # c2-o
   6   400.0000     1.3700 # o-c'
   7   615.3220     1.2300 # c'-o'
   8   322.8000     1.5000 # c'-c=
   9   655.2000     1.3300 # c=-c=
  10   361.6000     1.0900 # c=-h

Angle Coeffs # harmonic

   1    44.4000   110.0000 # c2-c3-h
   2    39.5000   106.4000 # h-c3-h
   3    46.6000   110.5000 # c3-c2-c2
   4    44.4000   110.0000 # c3-c2-h
   5    44.4000   110.0000 # c2-c2-h
   6    39.5000   106.4000 # h-c2-h
```

```
   7    46.6000   110.5000 # c2-c2-c2
   8    70.0000   109.5000 # c2-c2-o
   9    57.0000   109.5000 # o-c2-h
  10    60.0000   109.5000 # c2-o-c'
  11   145.0000   123.0000 # o-c'-o'
  12   122.0000   110.0000 # o-c'-c=
  13    50.0000   120.0000 # o'-c'-c=
  14    36.2000   122.3000 # c'-c=-c=
  15    36.2000   120.0000 # c'-c=-h
  16    33.8000   121.2000 # c=-c=-h
  17    37.5000   120.0000 # h-c=-h

Dihedral Coeffs # harmonic

  1     0.1581   1   3 # h-c3-c2-c2
  2     0.1581   1   3 # h-c3-c2-h
  3     0.1581   1   3 # c3-c2-c2-c2
  4     0.1581   1   3 # c3-c2-c2-h
  5     0.1581   1   3 # c2-c2-c2-h
  6     0.1581   1   3 # h-c2-c2-h
  7     0.1581   1   3 # c2-c2-c2-c2
  8     0.1581   1   3 # c2-c2-c2-o
  9     0.1581   1   3 # o-c2-c2-h
 10     0.1300   1   3 # c2-c2-o-c'
 11     0.1300   1   3 # h-c2-o-c'
 12     2.2500  -1   2 # c2-o-c'-o'
 13     2.2500  -1   2 # c2-o-c'-c=
 14     0.4500  -1   2 # o-c'-c=-c=
 15     0.4500  -1   2 # o-c'-c=-h
 16     0.4500  -1   2 # o'-c'-c=-c=
 17     0.4500  -1   2 # o'-c'-c=-h
 18     4.0750  -1   2 # c'-c=-c=-h
 19     4.0750  -1   2 # h-c=-c=-h

Improper Coeffs # cvff

    1    10.0000  -1   2 # o-c'-o'-c=
    2    11.1000  -1   2 # c'-c=-c=-h
    3    11.1000  -1   2 # c=-c=-h-h

Atoms # full

      1      1   1 -0.300000    -7.248175421     1.027165769    -
1.105747596   0   0   0 # c3
      2      1   2 -0.200000    -6.181919760     0.284273998    -
0.289269715   0   0   0 # c2
      3      1   2 -0.200000    -5.508568708     1.205917106
0.746154767   0   0   0 # c2
      4      1   2 -0.200000    -4.461012522     0.504221402
1.635490733   0   0   0 # c2
      5      1   2 -0.200000    -3.150686702     0.148111311
0.902706792   0   0   0 # c2
```

6 1 2 -0.050000 -2.102296566 -0.492994842
1.829716654 0 0 0 # c2
7 1 3 -0.180000 -0.912774083 -0.857728877
1.079999791 0 0 0 # o
8 1 4 0.410000 0.099809803 0.080474803
0.903905907 0 0 0 # c'
9 1 5 -0.380000 0.096942660 1.227518019
1.363489217 0 0 0 # o'
10 1 6 -0.100000 1.221883200 -0.491290818
0.063031455 0 0 0 # c=
11 1 6 -0.200000 2.333426374 0.157441140 -
0.297680242 0 0 0 # c=
12 1 7 0.100000 -7.725982483 0.359081609 -
1.846226820 0 0 0 # h
13 1 7 0.100000 -6.817866004 1.877924261 -
1.667293163 0 0 0 # h
14 1 7 0.100000 -8.054717730 1.428813682 -
0.463738904 0 0 0 # h
15 1 7 0.100000 -5.424379520 -0.138935891 -
0.976770864 0 0 0 # h
16 1 7 0.100000 -6.641889291 -0.585975381
0.218670502 0 0 0 # h
17 1 7 0.100000 -6.290117638 1.637287377
1.401835307 0 0 0 # h
18 1 7 0.100000 -5.048859488 2.078568185
0.241176698 0 0 0 # h
19 1 7 0.100000 -4.905343947 -0.399941142
2.096500840 0 0 0 # h
20 1 7 0.100000 -4.224868591 1.173245222
2.486419559 0 0 0 # h
21 1 7 0.100000 -2.718565906 1.058035106
0.442254124 0 0 0 # h
22 1 7 0.100000 -3.356103221 -0.539730237
0.060913172 0 0 0 # h
23 1 7 0.100000 -2.520852872 -1.409123242
2.288125103 0 0 0 # h
24 1 7 0.100000 -1.858626426 0.173989364
2.682419172 0 0 0 # h
25 1 7 0.100000 1.102099158 -1.521477635 -
0.272945661 0 0 0 # h
26 1 7 0.100000 3.089615901 -0.337515611 -
0.908303574 0 0 0 # h
27 1 7 0.100000 2.507812609 1.191124500
0.009028123 0 0 0 # h

Bonds

1 1 1 2
2 2 1 12
3 2 1 13
4 2 1 14
5 3 2 3

6	4	2	15
7	4	2	16
8	3	3	4
9	4	3	17
10	4	3	18
11	3	4	5
12	4	4	19
13	4	4	20
14	3	5	6
15	4	5	21
16	4	5	22
17	5	6	7
18	4	6	23
19	4	6	24
20	6	7	8
21	7	8	9
22	8	8	10
23	9	10	11
24	10	10	25
25	10	11	26
26	10	11	27

Angles

1	1	2	1	12
2	1	2	1	13
3	1	2	1	14
4	2	12	1	13
5	2	12	1	14
6	2	13	1	14
7	3	1	2	3
8	4	1	2	15
9	4	1	2	16
10	5	3	2	15
11	5	3	2	16
12	6	15	2	16
13	7	2	3	4
14	5	2	3	17
15	5	2	3	18
16	5	4	3	17
17	5	4	3	18
18	6	17	3	18
19	7	3	4	5
20	5	3	4	19
21	5	3	4	20
22	5	5	4	19
23	5	5	4	20
24	6	19	4	20
25	7	4	5	6
26	5	4	5	21
27	5	4	5	22
28	5	6	5	21

29	5	6	5	22
30	6	21	5	22
31	8	5	6	7
32	5	5	6	23
33	5	5	6	24
34	9	7	6	23
35	9	7	6	24
36	6	23	6	24
37	10	6	7	8
38	11	7	8	9
39	12	7	8	10
40	13	9	8	10
41	14	8	10	11
42	15	8	10	25
43	16	11	10	25
44	16	10	11	26
45	16	10	11	27
46	17	26	11	27

Dihedrals

1	1	12	1	2	3
2	2	12	1	2	15
3	2	12	1	2	16
4	1	13	1	2	3
5	2	13	1	2	15
6	2	13	1	2	16
7	1	14	1	2	3
8	2	14	1	2	15
9	2	14	1	2	16
10	3	1	2	3	4
11	4	1	2	3	17
12	4	1	2	3	18
13	5	4	3	2	15
14	6	15	2	3	17
15	6	15	2	3	18
16	5	4	3	2	16
17	6	16	2	3	17
18	6	16	2	3	18
19	7	2	3	4	5
20	5	2	3	4	19
21	5	2	3	4	20
22	5	5	4	3	17
23	6	17	3	4	19
24	6	17	3	4	20
25	5	5	4	3	18
26	6	18	3	4	19
27	6	18	3	4	20
28	7	3	4	5	6
29	5	3	4	5	21
30	5	3	4	5	22
31	5	6	5	4	19

```
    32   6     19      4      5     21
    33   6     19      4      5     22
    34   5      6      5      4     20
    35   6     20      4      5     21
    36   6     20      4      5     22
    37   8      4      5      6      7
    38   5      4      5      6     23
    39   5      4      5      6     24
    40   9      7      6      5     21
    41   6     21      5      6     23
    42   6     21      5      6     24
    43   9      7      6      5     22
    44   6     22      5      6     23
    45   6     22      5      6     24
    46  10      5      6      7      8
    47  11     23      6      7      8
    48  11     24      6      7      8
    49  12      6      7      8      9
    50  13      6      7      8     10
    51  14      7      8     10     11
    52  15      7      8     10     25
    53  16      9      8     10     11
    54  17      9      8     10     25
    55  18      8     10     11     26
    56  18      8     10     11     27
    57  19     25     10     11     26
    58  19     25     10     11     27

Impropers

     1   1      7      8      9     10
     2   2      8     10     11     25
     3   3     10     11     26     27
```

Please refer to LAMMPS documentation for the data file syntax.

Step 9: Running MD in LAMMPS: A sample LAMMPS script is shown below to setup a MD run with the hexyl acrylate molecule. Please refer to LAMMPS documentation for syntax and command parameter explanations.

```
log hexyl_acrylate.log

units real
atom_style full
boundary   p

pair_style lj/cut/coul/cut 9.5
```

```
      bond_style      harmonic
      angle_style     harmonic
      dihedral_style  harmonic
      improper_style  cvff

      special_bonds lj/coul 0.0 0.0 1.0

      read_data nhexyl_acrylate_monomer.data

      dump Datom all hexyl_acrylate.xyz

      thermo_style custom step temp etotal press lx ly lz xy xz yz xlo
xhi ylo yhi zlo zhi vol pxx pyy pzz pxy pxz pyz
      thermo  1
      run 0

      min_style  cg    # cg is default
      minimize   1e-5  1e-6  2000  2000  #etol ftol maxiter maxeval


      velocity        all create 298.0 4337 dist gaussian
      neighbor        2.0 bin
      neigh_modify delay 5

      variable pr_npt equal press

      fix Fnpt all npt temp 298.0 298.0 100.0 iso ${pr_npt} 1.0 1000.0

      timestep 3.0

run 100
```

Prior to running MD in LAMMPS however, the system energies should be checked against MS values to ensure correct import. This can, for example, be done by selecting the various bonded and non-bonded energies for output in the thermo_style command as shown below (`etotal, evdwl, ecoul, epair, ebond, eangle, edihed, eimp`) and comparing term by term with MS output for the system in this exact same configuration.

```
thermo_style custom step temp etotal evdwl, ecoul, epair, ebond, eangle,
edihed, eimp, press lx ly lz
      thermo  1
      run 0
```

Please note that the terms differ between MS and LAMMPS. Dihedral is called torsion and improper is called out-of-plane in MS respectively. The *run 0* command enables the system energies to be output as imported, before the atoms are moved by timestepping.
